# The Effects and Mechanisms of Pomegranate on Regulating Lipid Metabolism

**DOI:** 10.1002/fsn3.71789

**Published:** 2026-04-15

**Authors:** Hao Wang, Jingtao Liu, Xin Dong, Tiantian Shi, Xuena Zhang, Yide He, Shaonan Hu, Yuewu Wang, Jian Zheng, Peifeng Xue

**Affiliations:** ^1^ Department of Pharmacy Inner Mongolia Medical University Hohhot China; ^2^ National Institutes for Food and Drug Control Beijing China

**Keywords:** hyperlipidemia, metabolomics, pomegranate, transcriptomics

## Abstract

Hyperlipidemia is a major risk factor for cardiovascular disease, yet effective dietary interventions remain underexplored. Pomegranate (POG) has been recognized for its potential lipid‐lowering effects, but its underlying mechanisms require further investigation. To elucidate the therapeutic mechanism of POG in high‐fat diet‐induced hyperlipidemic rats, a comprehensive strategy combining metabolomics and transcriptomics analyses was implemented. Here, we demonstrate that POG exerts hypolipidemic effects through modulation of lipid metabolism and inflammatory pathways. The findings revealed that POG administration significantly reduces body weight gain, liver weight, and adipose tissue accumulation, while alleviating hepatic steatosis. These metabolic improvements were accompanied by ameliorated lipid metabolism, manifested through significant reductions in serum triglycerides, total cholesterol, and low‐density lipoprotein cholesterol, with concurrent elevation of high‐density lipoprotein cholesterol. Metabolomic analysis reveals that POG treatment significantly alters sphingolipid, pyrimidine, and arachidonic acid metabolism, highlighting its role in lipid homeostasis. Furthermore, transcriptomic profiling identifies 440 differentially expressed genes enriched in lipid metabolism and inflammatory pathways, including lipid and atherosclerosis, NF‐κB, and TNF signaling pathways. These findings provide mechanistic insights into the regulatory effects of POG on lipid metabolism and inflammation, rendering it conducive to incorporation into functional foods or pharmaceutical applications.

## Graphical abstract

This study established a high‐fat diet‐induced hyperlipidemia rat model to investigate the lipid‐lowering effects of pomegranate. Pomegranate intervention significantly reduced serum total cholesterol (TC), triglyceride (TG), and low‐density lipoprotein cholesterol (LDL‐C) levels, while alleviating hepatic steatosis by decreasing liver weight and fat content. Serum metabolomics and liver transcriptomics analyses were performed to explore the underlying molecular mechanisms, revealing the regulatory effects of pomegranate on metabolic profiles and gene expression in hyperlipidemic rats.

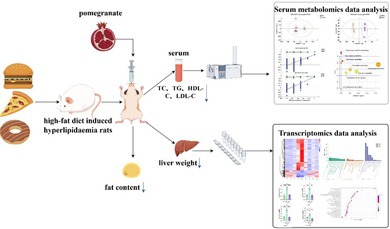

**Abbreviations**
ALT: alanine transaminase
AST: aspartate transaminase
BW: body weight
DBIL: direct bilirubin
H&E: hematoxylin and eosin
HDL‐C: high‐density lipoprotein cholesterol
HFD: high‐fat diet
HLP: hyperlipidemia
LDL‐C: low‐density lipoprotein cholesterol
NC: normal control
OPLS‐DA: orthogonal partial least squares discriminant analysis
PCA: unsupervised principal component analysis
POG: pomegranate
QC: quality control
RT‐qPCR: real‐time quantitative polymerase chain reaction
SD: standard deviation
SIM: simvastatin
SPF: specific pathogen‐free
TBIL: total bilirubin
TC: total cholesterol
TG: triglycerides

## 1 Introduction

Hyperlipidemia (HLP) is a prevalent cardiovascular condition categorized as primary or secondary HLP, primarily resulting from prolonged consumption of high‐fat, high‐calorie diets that induce metabolic dysregulation and localized lipid accumulation, and is particularly prevalent among the elderly population (Centers for Disease Control and Prevention 2011; Moghadam et al. 2020; Zhang et al. 2021). HLP is a metabolic disorder characterized by dysregulated lipid metabolism, leading to elevated serum lipid and lipoprotein profiles, including increased total cholesterol (TC) and triglyceride (TG) levels as well as decreased high‐density lipoprotein cholesterol (HDL‐C) levels. This aberrant lipid metabolism contributes to excessive fat accumulation in various organs, thereby increasing the risk of obesity and its associated complications, including fatty liver disease, coronary heart disease, type 2 diabetes mellitus, atherosclerosis, stroke, and myocardial infarction (Mikołajczak et al. 2021; She et al. 2022; Temu et al. 2021). Given the rapidly rising incidence of HLP, it has become a major global health concern. According to the World Health Organization, approximately 422 million adults worldwide are affected by HLP, accounting for 13.6% of the global adult population. In China, the prevalence of HLP continues to escalate annually, positioning it as a critical public health issue (Fan et al. 2018). The current pharmacological treatments of HLP primarily include statins (e.g., simvastatin, lovastatin, atorvastatin, pravastatin, and fluvastatin) and fibrates, which are widely prescribed to lower lipid levels. However, these agents often exhibit adverse effects such as myalgia, muscle weakness, hepatotoxicity, and gastrointestinal disturbances, in addition to concerns regarding long‐term dependency (Lu et al. 2023; Ouwens et al. 2015). Consequently, there is an urgent need for natural, low‐toxicity therapeutic alternatives that can effectively manage HLP with minimal side effects.

*Punica granatum* L., commonly known as pomegranate (POG), belongs to the Lythraceae family and is the fruit of a deciduous shrub or tree native to Central Asia. It is widely cultivated in various regions of China, including Shanxi, Henan, Xinjiang, Anhui, and others. Revered as a “precious fruit,” POG is not only nutrient‐rich but also a natural health supplement with dual pharmaceutical and culinary properties. It has been attributed various pharmacological activities, including antioxidant, antiaging, detoxifying, and Qi‐regulating properties.

A growing body of research has demonstrated the hypolipidemic and metabolic regulatory effects of POG. Studies have shown that pomegranate‐derived polyphenols, including ellagic acid and punicalagin, exert significant lipid‐lowering effects by modulating lipid metabolism and inflammation (Hou et al. 2019; Jiao et al. 2024). For instance, Barati Boldaji et al. (2020) reported that POG juice significantly reduced serum lipid levels in hyperlipidemic patients, suggesting its potential role as a functional dietary intervention. Furthermore, POG was found to mitigate diabetes and obesity‐associated fatty liver by activating hepatic fatty acid oxidation pathways (Xu et al. 2009). Additionally, polyphenolic compounds in POG have been shown to improve insulin resistance and lower blood glucose levels, highlighting their potential benefits in managing metabolic disorders (Sun, Zhou, and Wang 2019). Beyond clinical and in vivo studies, in vitro experiments have further elucidated the molecular mechanisms underlying POG's hypolipidemic effects. Research has demonstrated that POG polyphenols exert anti‐inflammatory effects by modulating TLR4‐NF‐κB signaling pathway (Li, Kouye, et al. 2022). These studies provide strong scientific evidence for the application of POG in the prevention and treatment of metabolic diseases. However, the mechanism of its hypolipidemic action remains to be fully elucidated owing to the complex interactions between its multiple components and targets.

To address this gap, our study employed a HFD‐induced HLP rat model combined with integrated metabolomic and transcriptomic analyses. We assessed the impact of POG supplementation on body weight, serum lipid profiles, organ weight ratios, and liver histopathology, and explored its effects on key metabolic and inflammatory pathways. This multi‐omics approach not only reinforces the hypolipidemic potential of POG but also provides novel mechanistic insights that may facilitate its future application as a safe and effective dietary intervention for managing HLP.

## 2 Materials and Methods

### 2.1 Experimental Animals and Reagents

The reporting of animal experiments follows the recommendations in the ARRIVE guidelines. Forty‐eight male SD rats, weighing between 200 and 220 g, were obtained from specific pathogen‐free (SPF) (Beijing) Biotechnology Company (license number: SCXK (Beijing) 2019‐0010). The animals were housed in a SPF facility at the Experimental Animal Center of Inner Mongolia Medical University, where they were maintained under standardized conditions. The rats were kept in a 12‐h light–dark cycle, with temperatures ranging from 20°C to 26°C and humidity levels of 40%–60%. The animals were provided with UV‐sterilized food and water, and their cages were cleaned weekly. All animal experiments were approved by the Ethics Committee for Animal Experiments of Inner Mongolia Medical University (No. YKD202101184).

The dried fruit of *Punica granatum* L. was obtained from Rende Xing Medicine Co. Ltd. in Anhui Province (batch number: 2203001) and authenticated by Professor Xue Peifeng. Each component content in pomegranate: punicalagin is 9.34 mg/g; gallic acid is 0.15 mg/g; ellagic acid is 3.79 mg/g. It was measured by high performance liquid chromatography (HPLC), as shown in Figures S1–S4. The dried fruit was ground into a fine powder (over 40 mesh screens) and dissolved in physiological saline before use. Simvastatin (SIM) (batch number: U024517) was purchased from Merck Sharp & Dohme in Hangzhou. Carboxymethyl cellulose sodium (batch number: D2115187) was obtained from Beijing Lanjie Technology Co. Ltd., while 4% paraformaldehyde (batch number: CR2207058) was purchased from Wuhan Saiwei Biotechnology Co. Ltd. The following test kits were also obtained: TC test kits (batch number: E20230904‐6688), TG test kits (batch number: E20230904‐6689), HDL‐C test kits (batch number: E20230904‐6690), LDL‐C test kits (batch number: E20230904‐6691), alanine transaminase (ALT) test kits (batch number: E20230904‐6666), aspartate transaminase (AST) test kits (batch number: E20230904‐6667), direct bilirubin (DBIL) test kits (batch number: E20230904‐6671), and total bilirubin (TBIL) test kits (batch number: E20230904‐6670) from Nanjing Built Biological Engineering Institute. The high‐fat feed (HFD) (58.6% basal feed + 15% swine oil + 20% sucrose + 5% casein + 1.2% cholesterol + sodium cholate) was purchased from Xiaoshu Youtai (Beijing) Biotechnology Co. Ltd. Additionally, hematoxylin and eosin (H&E) Staining Kit (G1003) was obtained from Wuhan Servier Biotechnology Co. Ltd., and TRIzol total RNA extraction reagent (Invitrogen TRIZOL Reagent, catalog number: cat. no. 15596‐018) was used for RNA extraction.

### 2.2 Animal Experiment and Sample Collection

Forty‐eight SPF‐grade SD rats were first subjected to a 1‐week acclimatization period. Randomization was performed by an independent investigator who assigned the rats to six groups. This investigator did not participate in subsequent animal husbandry, sample collection, or data analysis to avoid selection bias. The normal control (NC) group, fed with normal control chow, the HFD group receiving an HFD diet, the low‐dose POG group (consisting of HFD‐fed rats treated with POG at 0.42 g/kg body weight [BW], POG‐L), the medium‐dose POG group (HFD‐fed rats treated with POG at 0.84 g/kg BW, POG‐M), the high‐dose POG group (HFD‐fed rats treated with POG at 1.68 g/kg BW, POG‐H), and the SIM group (HFD‐fed rats treated with SIM at 4.2 mg/kg BW). The doses of POG were calculated based on the recommended dosages from the “Mongolian Medicine Volume of Traditional Chinese Medicine,” and the dose of SIM was determined according to clinical recommendations (Zhang et al. 2020). The equivalent doses for humans and animals were calculated using the body surface area method, with the daily dose for rats being 6.3 times that of an adult human. All rats, except for the NC group, were fed a HFD for 8 weeks, followed by daily intragastric administration of the respective treatments for another 8 weeks. The NC group received a standard chow diet for 8 weeks, followed by intragastric administration of an equivalent volume of saline. Daily weight increase, food consumption, and water intake were then tracked by two independent blinded operators every day.

Animals were fasted for 24 h prior to induction of anesthesia and had free access to water to minimize the risk of aspiration. Pentobarbital sodium solution (40 mg/kg) was given by intraperitoneal injection to induce anesthesia. Blood was collected from the abdominal aorta into non‐anticoagulated tubes and centrifuged at 3500 rpm for 10 min at 4°C. The supernatant was then stored at −80°C for further analysis. Following blood collection, the liver was excised immediately, with surface fat and connective tissue carefully removed on ice. The tissue was rinsed with ice‐cold physiological saline, blotted dry with absorbent paper, and weighed. A portion of the triangular lobe was rapidly frozen in liquid nitrogen and stored in a cryotube. The left lateral lobe was fixed in 4% paraformaldehyde, while the remaining liver tissue was wrapped in foil and immediately frozen in liquid nitrogen. Additionally, white adipose tissue was collected, washed with saline, blotted dry to remove bloodstains, and weighed.

### 2.3 Serum Biochemical Analysis

Serum samples were collected from the centrifuged blood and the supernatant was used for biochemical analysis. According to the manufacturer's instructions, the levels of TC, TG, HDL‐C, LDL‐C, ALT, AST, DBIL, and TBIL in the serum samples were measured using commercial assay kits.

### 2.4 Histopathological Analysis

For histopathological examination, liver tissues were fixed in 10% formaldehyde for 24 h. The fixed liver tissues were then sectioned in the same direction and dehydrated through a series of ethanol solutions (70%, 80%, 95%, and 100%) to facilitate paraffin embedding. The dehydrated liver tissues were then embedded in paraffin wax. The paraffin‐embedded liver tissues were sectioned and deparaffinized using xylene, followed by a series of ethanol solutions (100%, 95%, 80%, and 70%) to remove residual wax. The sections were then washed with water and stained with hematoxylin and eosin to display the tissue structure and cellular morphology. After staining, the sections were washed with distilled water. Finally, the sections were dehydrated through a series of ethanol solutions (70%, 80%, 95%, and 100%) and xylene, and mounted with neutral gum for microscopic observation and photography (Shen et al. 2024).

### 2.5 Metabolomics Analysis

Blood serum samples from each group were thawed at 4°C, and 10 μL of each sample was mixed well and transferred to a dry, sterile centrifuge tube labeled as the quality control (QC) sample. Additionally, 200 μL of each serum sample was transferred to a dry, sterile centrifuge tube. A threefold volume of methanol was added to the QC sample and each serum sample, vortex‐mixed for 5 min, and then centrifuged at 14,000 rpm for 10 min at 4°C. The supernatant was collected, and the resulting pellets were dried and redissolved in 200 μL of methanol, vortex‐mixed for 5 min, and centrifuged at 14,000 rpm for 10 min. The supernatant was transferred to an autosampler vial for UPLC‐Q‐Exactive‐MS analysis. The UPLC‐Q‐Exactive‐MS analysis was performed using a Waters ACQUITY UPLC HSS T3 column (2.1 × 50 mm, 1.8 μm). The mobile phase consisted of 0.1% formic acid in water (A) and methanol (B) with a gradient elution: 0–1.7 min, 5%–15% B; 1.7–8.0 min, 15%–55% B; 8.0–12.0 min, 55%–75% B; 12.0–14.0 min, 5% B; 14.0–16.0 min, 75%–100% B; 16.0–17.0 min, 100% B; 17.0–18.0 min, 0%–5% B; 18.0–20.0 min, 5% B. The column temperature was set at 35°C, and the flow rate was 0.4 mL/min. The injection volume was 5 μL. The resulting chromatograms were analyzed using Discoverer Compound 3.3 software for peak identification, peak filtering, peak extraction, and peak area calculation. The resulting data were exported to SIMCA 14.1 software for principal component analysis (PCA) to examine the clustering of samples within each group and the metabolic profile changes between groups. The models were then subjected to orthogonal partial least squares discriminant analysis (OPLS‐DA) to identify the variables with VIP > 1. The resulting metabolites with VIP > 1 and *p* < 0.05 were selected as potential biomarkers. The identified biomarkers were further validated using PubChem (https://pubchem.ncbi.nlm.nih.gov/) and the Human Metabolome Database (HMDB) (https://hmdb.ca/). Finally, the identified biomarkers were analyzed using MetaboAnalyst 6.0 (https://www.metaboanalyst.ca/) to identify the affected metabolic pathways (Pang et al. 2024).

### 2.6 Transcriptomics Profiling

Total RNA was isolated from rat liver tissue, and its purity and concentration were evaluated using a Nanodrop2000 microvolume spectrophotometer. The integrity of the RNA sample was assessed using the Labchip GX touch microfluidic chip system. Following quality control, oligo (dT) magnetic beads were used to enrich for eukaryotic mRNA. The mRNA was then fragmented and used as a template for synthesizing the first strand of cDNA using a six‐base random primer. The second strand of cDNA was synthesized in the presence of buffers, dNTPs, DNA polymerase I, and RNase H, followed by purification. The double‐stranded cDNA underwent end repair, addition of a base A, and sequencing adapters, and was then size‐selected to recover approximately 350 bp fragments. These fragments were amplified by PCR to create a cDNA library. The cDNA synthesis was initially quantitated using the Qubit 3.0, and the library fragment size was preliminarily assessed using the Agilent 2100 system. The libraries were then pooled based on their effective concentrations and the target data volume required for sequencing. The pooled libraries underwent sequencing, generating 150 bp paired‐end reads. The sequencing principle employed was Sequencing by Synthesis (SBS), which utilizes fluorescently labeled dNTPs, DNA polymerase, and adapter primers for amplification. As each fluorescently labeled dNTP was incorporated during the extension of the complementary strands, it released fluorescence that was captured by the sequencer. Computer software translated these light signals into sequencing peaks, thereby obtaining the sequence information of the target fragments.

Differential gene expression analysis was performed using the DESeq2 software package for samples with biological duplicates. This software provides a statistical framework for identifying differentially expressed genes in numerical gene expression data using models based on negative binomial distributions. To control for false discovery rates, the resulting *p* values were adjusted using the method of Benjamini and Hochberg. Genes with adjusted *p* ≤ 0.05 were considered to be differentially expressed (Li, Zand, et al. 2022). For samples without biological replicates, differential gene expression analysis was performed using the DESeq software package. Functional enrichment analysis of differentially expressed genes was performed using the DAVID software package, which identified Gene Ontology (GO) terms and Kyoto Encyclopedia of Genes and Genomes (KEGG) pathways that were significantly enriched (*p* < 0.05).

### 2.7 Real‐Time Quantitative PCR Determination

An RNA extraction kit (Invitrogen TRIZOL Reagent) was employed to isolate total RNA from the liver tissue for subsequent analysis. Using a reverse transcription reaction method, the isolated RNA was reverse transcribed into complementary DNAs. Using a PCR apparatus, templates and primers were combined with 2× Universal Blue SYBR Green qPCR Master Mix and amplified. Using a fluorescent quantitative PCR device, fluorescence signals during the PCR reaction were tracked in real time. The results were relative quantified using the ΔΔCT technique. RNA levels were normalized with β‐actin. Primers unique to the genes listed in Table 1 were used to perform real‐time fluorescence polymerase chain reaction.

**TABLE 1:** Real‐time PCR primer sequences.

Gene	Forward primer sequence (5′–3′)	Reverse primer sequence (5′–3′)
*β‐actin*	TGCTATGTTGCCCTAGACTTCG	GTTGGCATAGAGGTCTTTACGG
*LTB*	CTTCCTGCTGCCCACCTCATA	GATACCCGACATGGCAGTAGAGATA
*TNFSF13B*	TGTTCCATGGCTTCTCAGCTTTA	GAACCTGGCTGTAGATGAAGAAAT
*RELB*	TAACAACTTGGGCATCCAGTGT	GTTCTTCAGGGAGCCAGCATT
*BIRC3*	GCTGTGATGGTGGGCTAAGAT	GCTCAAGTAGATGAGGGTAACCAG

### 2.8 Statistical Analysis

The experimental data were statistically analyzed and collated using SPSS 26.0 software. The results were expressed as “mean ± standard deviation (SD).” One‐way ANOVA followed by post hoc test was used for multiple comparisons, with *p* < 0.05 considered statistically significant**. For transcriptomic and metabolomic analyses, **differentially expressed genes were defined with |log_2_ (fold change)| > 1.0 and adjusted *p* value < 0.05, while differential metabolites were identified based on variable importance in the projection (VIP) > 1.0, fold change > 1.2 or < 0.83, and *p* < 0.05. GraphPad Prism 9.0 was used for data visualization and figure construction.

## 3 Results

### 3.1 Identification of Active Compounds in POG

In this study, the characteristic chemical profile of POG was systematically analyzed using high performance liquid chromatography‐mass spectrometry (HPLC‐MS/MS) combined with multistage mass spectrometry cleavage patterns. The results showed that the chemical diversity is mainly reflected in the following four major groups of compounds: tannins and their hydrolysis products, flavonoids, ellagic acid derivatives, and fatty acids (Table 2).

**TABLE 2:** Compounds in POG.

No.	RT (min)	Ionization mode	Molecular formula	Mass accuracy (ppm)	Fragmentations (m/z)	Compounds identified
1	3.15	[M − H]^−^	C_20_H_20_O_14_	2.025	331.0670, 331.0568, 169.0131, 125.0230	1,6‐Di‐O‐galloyl‐beta‐D‐glucose
2	3.51	[M − H]^−^	C_20_H_20_O_14_	2.025	331.0670, 313.0568, 271.0465, 241.0343, 211.0240, 169.0131, 125.0230	1,6‐Di‐O‐galloyl‐beta‐D‐glucose
3	3.90	[M − H]^−^	C_15_H_14_O_7_	1.511	261.0770, 221.0445, 219.0653, 179.0343, 137.0230	Gallocatechin
4	3.90	[M − H]^−^	C_15_H_14_O_7_	1.511	261.0770, 221.0445, 219.0653, 179.0343, 137.0230, 125.0231	(−)‐Epigallocatechin
5	5.68	[M − H]^−^	C_7_H_6_O_5_	0.12	125.0230	Gallic acid
6	6.03	[M − H]^−^	C_7_H_6_O_4_	0.097	109.0281	Protocatechuic acid
7	10.10	[M − H]^−^	C_48_H_28_O_30_	−2.193	781.0521, 600.9896, 575.0106, 300.9987, 270.9881	Punicalagin
8	11.41	[M − H]^−^	C_34_H_24_O_22_	−0.841	481.0629, 300.9989, 275.0197, 229.0144	Pedunculagin
9	14.74	[M − H]^−^	C_15_H_14_O_6_	3.755	245.0814, 227.0703, 205.0500, 207.0603, 187.0389, 179.0335, 161.0593, 137.0231, 125.0230, 109.0281	Cianidanol
10	14.74	[M − H]^−^	C_15_H_14_O_6_	3.305	245.0814, 227.0703, 205.0500, 203.0703, 187.0389, 179.0335, 161.0593, 137.0321, 125.0230, 109.0281	L‐Epicatechin
11	15.08	[M − H]^−^	C_8_H_8_O_5_	1.312	168.0054, 139.0024, 124.0152	Methyl gallate
12	19.34	[M − H]^−^	C_34_H_26_O_22_	0.766	300.9989, 275.0196, 169.0131, 125.0229	Tellimagradin I
13	19.34	[M − H]^−^	C_34_H_26_O_22_	0.766	615.0578, 300.9989, 275.0196, 169.0131, 125.0229	Tellimagradin I
14	22.78	[M − H]^−^	C_21_H_22_O_11_	1.252	287.0560, 259.0607, 125.0229	3,5‐Dihydroxy‐2‐(4‐hydroxyphenyl)‐7‐[3,4,5‐trihydroxy‐6‐(hydroxy‐methyl) oxan‐2‐yl]oxy‐2,3‐dihydrochromen‐4‐one
15	23.18	[M − H]^−^	C_27_H_22_O_18_	1.106	463.0520, 300.9989, 275.0195, 229.0133, 169.0131	Corilagin
16	24.60	[M − H]^−^	C_41_H_28_O_26_	0.336	633.0753, 300.9988, 257.0093, 169.0124	Casuarinin
17	24.92	[M − H]^−^	C_27_H_24_O_18_	1.543	483.0780, 465.0670, 331.0675, 313.0562, 211.0244, 193.0132, 169.0131, 151.0026, 125.0230	1,3,6‐Tri‐o‐galloylglucose
18	27.36	[M − H]^−^	C_28_H_10_O_16_	1.547	300.9990, 298.9833, 270.9878	Gallagic acid dilactone
19	27.85	[M − H]^−^	C_21_H_20_O_13_	1.676	316.0222, 287.0179, 271.0243	Myricetin 3‐β‐D‐glucopyranoside
20	28.23	[M − H]^−^	C_34_H_24_O_22_	−0.062	481.0622, 300.9988, 275.0196, 229.0136	Pedunculagin
21	29.53	[M − H]^−^	C_34_H_26_O_22_	−0.635	617.0756, 463.0524, 300.9989, 275.0197, 169.0131, 125.0231	Tellimagradin I
22	30.08	[M − H]^−^	C_19_H_14_O_12_	2.35	300.9982	Ellagic acid pentoside
23	30.43	[M − H]^−^	C_19_H_14_O_12_	2.766	300.9984, 271.0610	Ellagic acid pentoside
24	31.17	[M − H]^−^	C_27_H_30_O_16_	1.919	301.0344, 300.0273, 151.0028	Rutin
25	32.14	[M − H]^−^	C_14_H_6_O_8_	3.543	283.9969, 257.0087, 229.0140	Ellagic acid
26	32.22	[M − H]^−^	C_21_H_20_O_12_	2.219	301.0340, 300.0275, 271.0245, 255.0292, 151.0026	Isoquercetin
27	33.38	[M − H]^−^	C_20_H_18_O_11_	4.646	301.0343, 271.0232, 255.0299, 178.9978, 151.0024	Quercetin‐3‐O‐arabinoside
28	34.13	[M − H]^−^	C_27_H_30_O_15_	2.248	285.0401, 255.0291, 151.0022	KaeMpferol‐3‐O‐rutinoside
29	35.97	[M − H]^−^	C_21_H_20_O_10_	2.382	268.0376	Apigenin 7‐glucoside
30	37.74	[M − H]^−^	C_21_H_20_O_10_	2.521	285.0399, 255.0294, 151.0025	Afzelin
31	39.95	[M − H]^−^	C_28_H_28_O_14_	2.688	481.0987, 313.0565, 273.0768, 169.0131, 167.0337	[(2R,3S,4S,5R,6S)‐6‐[3,5‐dihydroxy‐4‐[3‐(4‐hydroxyphenyl) pro‐panoyl]phenoxy]‐3,4,5‐trihydroxyoxan‐2‐yl]methyl 3,4,5‐trihyd‐roxybenzoate
32	40.45	[M − H]^−^	C_22_H_22_O_11_	4.19	313.0587, 211.0240, 169.0131, 125.0229	Cinnamoyl‐glucogallin
33	43.41	[M − H]^−^	C_15_H_14_O_5_	4.211	167.0339, 123.0438, 81.0332	Phloretin
34	44.21	[M − H]^−^	C_15_H_10_O_6_	3.317	151.0025	Kaempferol
35	47.10	[M − H]^−^	C_17_H_12_O_8_	2.817	328.0223, 312.9987, 269.9755	5,6‐Dihydroxy‐3′,4′,7‐trimethoxy‐flavone
36	64.49	[M − H]^−^	C_16_H_32_O_2_	3.343	255.2327	Palmitic acid
37	38.41	[M + H]+	C_21_ H_10_ O_13_	2.321	493.0085, 407.0031, 287.0175	Valoneic acid dilactone

### 3.2 Effect of POG on Lipid Homeostasis and Body Weight, Liver Function

**FIGURE 1:**
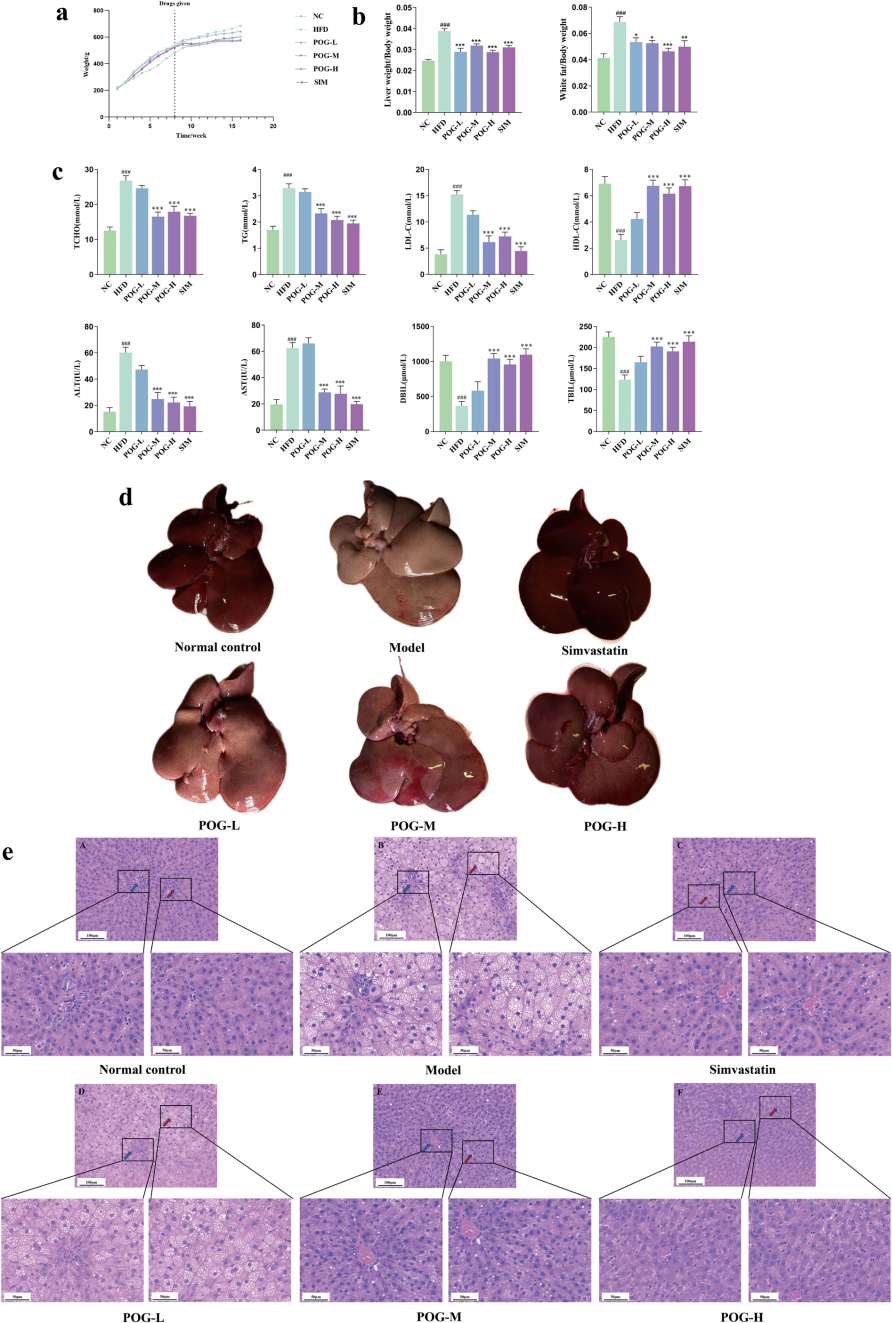
Pharmacodynamic evaluation of POG against HLP. (a) Body weight change of rats in each group during the experiment period. (b) “liver weight/body weight” and “white adipose tissue weight/body weight” of each group. (c) Comparison of serum biochemical indexes of rats in each group, compared with the blank group, ^#^ *p* < 0.05, ^##^ *p* < 0.01, ^###^ *p* < 0.001; compared with the model group *p < 0.05, **p < 0.01, ***p < 0.001. (d) Liver pictures of rats in each group. (e) Effect of POG on the liver samples, scale bar = 50 and 100 μm (red arrows: macrovesicular steatosis displacing hepatocyte nuclei; blue arrows: central vein).

After an 8‐week treatment period, the body weight of the HFD group rats was significantly higher than that of the blank‐control group rats. In contrast, the body weights of the treated groups were all lower than those of the HFD group rats (Figure 1a). One‐way ANOVA revealed a significant main effect of treatment for the liver weight/body weight ratio (*F*(5, 42) = 17.65, *p* < 0.0001) and white adipose tissue weight/body weight ratio (*F*(5, 42) = 7.769, *p* < 0.0001). Compared to the NC group, both ratios were significantly increased in the HFD group (*p* < 0.0001 for both), and all treatment groups showed significant reductions in these indices relative to the HFD group (all *p* < 0.01) (Figure 1b).

For serum biochemical parameters, one‐way ANOVA demonstrated significant main effects of treatment on total cholesterol (TC, *F*(5, 42) = 20.80, *p* < 0.0001), triglyceride (TG, *F*(5, 42) = 19.55, *p* < 0.0001), low‐density lipoprotein cholesterol (LDL‐C, *F*(5, 42) = 24.67, *p* < 0.0001), high‐density lipoprotein cholesterol (HDL‐C, *F*(5, 42) = 13.66, *p* < 0.0001), alanine aminotransferase (ALT, *F*(5, 42) = 21.33, *p* < 0.0001), aspartate aminotransferase (AST, *F*(5, 42) = 29.20, *p* < 0.0001), direct bilirubin (DBIL, *F*(5, 42) = 11.41, *p* < 0.0001), and total bilirubin (TBIL, *F*(5, 42) = 9.601, *p* < 0.0001). Post hoc analysis revealed that the HFD group exhibited significantly elevated levels of TC, TG, LDL‐C, ALT, and AST (all *p* < 0.0001) and reduced levels of HDL‐C, DBIL, and TBIL (all *p* < 0.0001) compared to the NC group, and the medium‐dose group and high‐dose group in the treatment group showed significant reductions in these indices relative to the HFD group (all *p* < 0.01) (Figure 1c).

### 3.3 Effect of POG on Liver Histopathology

As shown in the rat liver images, the control group exhibited soft hepatic texture with sharp edges and uniform dark‐red coloration, while the model group displayed hardened liver consistency, rounded margins, and earthy‐yellow discoloration. All treatment groups demonstrated varying degrees of amelioration in HLP‐induced hepatic damage (Figure 1d). Histological examination of liver tissue sections via H&E staining confirmed the successful establishment of the HFD model. In the NC group, liver tissue sections exhibited normal hepatocyte morphology, characterized by a radial arrangement around the central vein and intact cell boundaries, with no significant lipid droplet accumulation around the cells. In contrast, hepatocytes in the HFD group exhibited severe damage, as evidenced by disordered cellular arrangements, prominent vacuolated structures within cells, and increased lipid droplets in the surrounding area. Notably, treatment with various doses of POG and SIM improved liver cell lesions to varying degrees, with liver cells trending toward normalcy (Figure 1e).

### 3.4 Effect of POG on Serum Metabolic Profiles

#### 3.4.1 Serum Metabolomics Data Analysis

**FIGURE 2:**
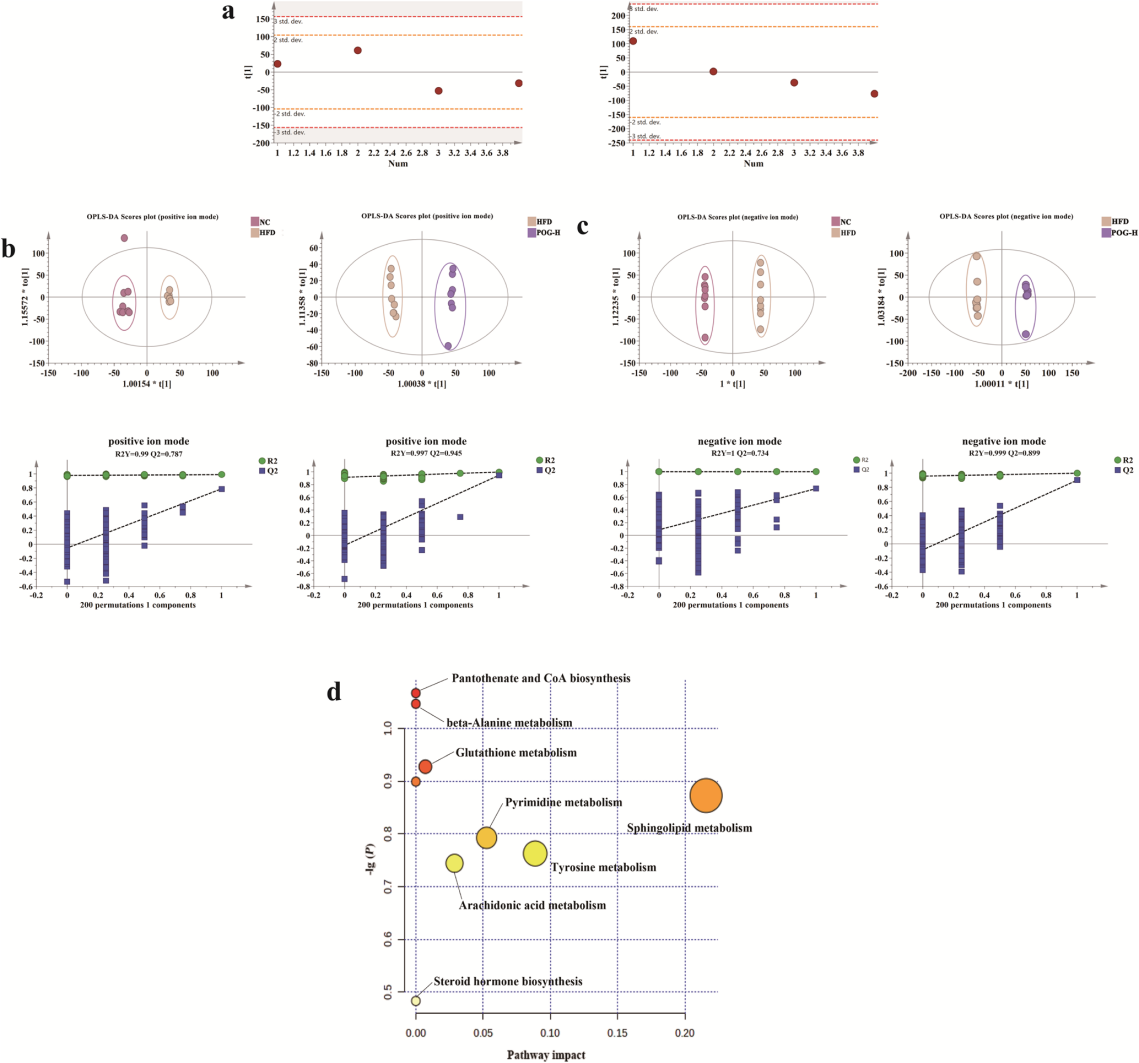
Serum metabolomics results of POG against HLP. (a) 1D distribution of QC in positive and negative ion modes. (b) Plot of OPLS‐DA and substitution test scores in positive and negative ion modes NC versus HFD. (c) Plot of OPLS‐DA and substitution test scores in positive and negative ion modes HFD versus POG. (d) POG improves metabolic pathways in HFD rats.

To ensure the reliability of our analytical methods and instruments, we employed an unsupervised principal component analysis (PCA) approach. Under this framework, all quality control (QC) samples fell within the ±2 standard deviations range on the one‐dimensional distribution map (Figure 2a), indicating the stability and repeatability of our experimental data throughout the analysis process. Subsequently, we applied orthogonal partial least squares discriminant analysis (OPLS‐DA) to investigate the serum metabolic profiles between groups and identify differential metabolites. The quality of the model was assessed using predictive parameters such as *R* ^2^ *Y* and *Q* ^2^, where higher values indicate a more stable and reliable model. A *Q* ^2^ value > 0.5 is considered effective, while a *Q* ^2^ value exceeding 0.9 is regarded as excellent. In both positive and negative ion modes, the serum metabolic profiles of the NC group and the HFD group rats were distinct, occupying separate quadrants. The *R* ^2^ *Y* and *Q* ^2^ values in positive ion mode were 0.99 and 0.787, respectively, and in negative ion mode were 1 and 0.706, respectively, indicating good model stability without overfitting and possessing satisfactory predictive capabilities. These results demonstrate that after 8 weeks of modeling, a HFD model was successfully established, and significant changes in metabolism were observed between the two groups of rats (Figure 2b). Furthermore, the serum metabolic profiles of the POG‐H group and the HFD group rats also showed clear differences in both positive and negative ion modes. The *R* ^2^ *Y* and *Q* ^2^ values in positive ion mode were 0.997 and 0.945, respectively, and in negative ion mode were 0.999 and 0.899, respectively, further confirming the model's stability and predictive ability. These findings suggest that after 8 weeks of POG treatment, significant alterations in the serum metabolic profile of HFD rats were observed (Figure 2c).

#### 3.4.2 Identification of the Biomarkers and Pathway Analysis

To identify potential differences between groups, we employed an orthogonal partial least squares discriminant analysis (OPLS‐DA) model, with variable importance in projection (VIP) > 1 and *p* < 0.05 as the screening criteria. This approach enabled the identification of 85 differential metabolites between the NC and HFD groups, which are considered endogenous biomarkers of the HFD model. Notably, upon administering POG treatment to rats, a total of 37 differential metabolites were observed to have been reversed (Table 3). These metabolites included pyrazolone H, noradrenaline, cholesteryl sulfate, glycocholic acid, tazarotene, ethylparaben, L‐carnitine, and 3,4‐dimethoxybenzeneamine, among others. To further elucidate the biochemical markers related to HLP regulated by POG, we utilized MetaboAnalyst 6.0 to analyze the 37 screened metabolic compounds. This analysis revealed a total of nine metabolic pathways (Figure 2d), including pantothenate and CoA biosynthesis, beta‐alanine metabolism, glutathione metabolism, pyrimidine metabolism, sphingolipid metabolism, tyrosine metabolism, arachidonic acid metabolism, steroid hormone biosynthesis, and lysine degradation. These findings provide insight into the metabolic mechanisms underlying the therapeutic effects of POG on HLP.

**TABLE 3:** Significant callback of differential metabolites.

No.	Metabolites	Formula	Ion mode	Precursor ions	ppm	NC vs. HFD		HFD vs. POG	
						VIP	Trend	VIP	Trend
1	1‐(14‐methylhexadecanoyl)pyrrolidine	C_21_H_41_NO	POS	[M + H]^+1^	−0.16	1.42101	↑	1.19726	↓
2	2‐[(5Z)‐5‐tetradecenyl]cyclobutanone	C_18_H_32_O	POS	[M + H]^+1^	0.36	1.51828	↑	1.33897	↓
3	DL‐Carnitine	C_7_H_15_NO_3_	POS	[M + H]^+1^	2.22	2.07537	↑	1.39925	↓
4	Triglyceride LOL, sn	C_57_H_100_O_6_	POS	[M + H]^+1^	−3.89	1.84002	↓	1.31755	↑
5	Ethylparaben	C_9_H_10_O_3_	POS	[M + H]^+1^	3.23	1.75989	↑	1.32871	↓
6	Limaprost	C_22_H_36_O_5_	POS	[M + Na]^+1^	−2.81	1.32169	↓	1.21391	↑
7	N‐(2‐hydroxyoctadecanoyl)‐hydroxysphinganine	C_36_H_73_NO_5_	POS	[M + H‐H^2^O]^+1^	−2.49	1.68709	↑	1.6147	↓
8	1‐Myristoleoyl‐2‐docosahexaenoyl‐sn‐glycero‐3‐phosphoethanolamine	C_41_H_68_NO_8_P	POS	[M + H]^+1^	−3.22	1.61772	↓	1.14554	↑
9	Fulvestrant	C_32_H_47_F_5_O_3_S	POS	[M + Na]^+1^	−4.22	1.61975	↓	1.1858	↑
10	Phenethyl octanoate	C_16_H_24_O_2_	POS	[M + H]^+1^	−4.59	1.88942	↓	1.06907	↑
11	Ibrutinib	C_25_H_24_N_6_O_2_	POS	[M + H]^+1^	−2.48	2.03033	↓	1.5548	↑
12	(−)‐Prostaglandin E2	C_20_H_32_O_5_	POS	[M + Na]^+1^	−4.12	1.70943	↓	1.28	↑
13	Melamine	C_3_H_6_N_6_	POS	[M + H]^+1^	3.93	1.18562	↓	1.30972	↑
14	3,4‐Dimethoxyphenethylamine	C_10_H_15_NO_2_	POS	[M + H]^+1^	2.1	1.73483	↓	1.1749	↑
15	1‐(1H‐Imidazo[4,5‐c]pyridin‐4‐yl)ethanone	C_8_H_7_N_3_O	POS	[M + H]^+1^	3.29	1.33975	↓	1.25797	↑
16	L‐Pyroglutamic acid	C_5_H_7_NO_3_	POS	[M + H]^+1^	4.09	1.86559	↑	1.69861	↓
17	Acrimarine H	C_30_H_27_NO_7_	NEG	[M − H]^−1^	2.49	2.43368	↓	1.57079	↑
18	Cholesterol sulfate	C_27_H_46_O_4_S	NEG	[M − H]^−1^	−1.03	1.60231	↑	1.80296	↓
19	Noradrenaline	C_8_H_11_NO_3_	NEG	[M − H + HAc]^−1^	−0.11	1.60851	↑	2.02701	↓
20	2‐Fucosyllactose	C_18_H_32_O_15_	NEG	[M − H]^−1^	4.59	2.34679	↓	1.38075	↑
21	3,4‐Dimethyl‐5‐pentyl‐2‐furanpentadecanoic acid	C_26_H_46_O_3_	NEG	[M − H]^−1^	1	1.73611	↓	1.85713	↑
22	Tazarotene	C_21_H_21_NO_2_S	NEG	[M − H]^−1^	1.93	1.90305	↓	1.26794	↑
23	Ceramide (d18:1/24:0)	C_42_H_83_NO_3_	NEG	[M − H]^−1^	−0.85	1.64248	↑	1.36233	↓
24	Ecdysterone	C_27_H_44_O_7_	NEG	[M − H]^−1^	2.04	1.4831	↑	1.26267	↓
25	Garcinone D	C_24_H_28_O_7_	NEG	[M − H]^−1^	−2.75	1.95408	↓	1.1546	↑
26	2,4‐dihydroxyheptadec‐16‐enyl acetate	C_19_H_36_O_4_	NEG	[M − H]^−1^	2.89	1.46908	↑	1.56937	↓
27	Rose bengal (photoactivated)	C_20_H_4_Cl_4_I_4_O_5_	NEG	[M − H]^−1^	4.28	1.78958	↑	1.45469	↓
28	MU‐Ac	C_12_H_10_O_4_	NEG	[M − H]^−1^	3.56	1.50473	↑	1.60309	↓
29	Uracil	C_4_H_4_N_2_O_2_	NEG	[M − H]^−1^	2.62	1.34927	↑	2.20326	↓
30	Fostamatinib	C_23_H_26_FN_6_O_9_P	NEG	[M − H]^−1^	0.89	1.64873	↑	1.26153	↓
31	2 (3H)‐Furanone, dihydro‐3, 4‐divanillyl—	C_20_H_22_O_6_	NEG	[M − H]^−1^	1.21	2.42258	↑	2.02565	↓
32	Phyllanthin	C_24_H_34_O_6_	NEG	[M − H]^−1^	−4.69	1.49582	↓	1.87287	↑
33	Glycochenodeoxycholic acid	C_26_H_43_NO_5_	NEG	[M − H]^−1^	0.49	1.38409	↑	1.15212	↓
34	Pyrogallol	C_6_H_6_O_3_	NEG	[M − H]^−1^	2.9	1.8254	↓	1.16593	↑
35	Ethyl 3‐(methylthio)propionate	C_6_H_12_O_2_S	NEG	[M − H]^−1^	−3.56	1.42906	↓	1.13847	↑
36	1‐nonanoic acid	C_9_H_18_O_2_	NEG	[M − H]^−1^	−5.4	1.45832	↑	1.27954	↓
37	(4Z,7Z,10Z,19Z)‐14‐[(4Z,7Z,10Z,13Z,16Z,19Z)‐4,7,10,13,16,19‐Docosahexaenoyloxy]‐4,7,10,12,16,19‐docosahexaenoic acid	C_44_H_62_O_4_	NEG	[M − H]^−1^	0.15	1.46672	↓	1.79449	↑

### 3.5 Effect of POG on Transcriptomics Analysis

**FIGURE 3:**
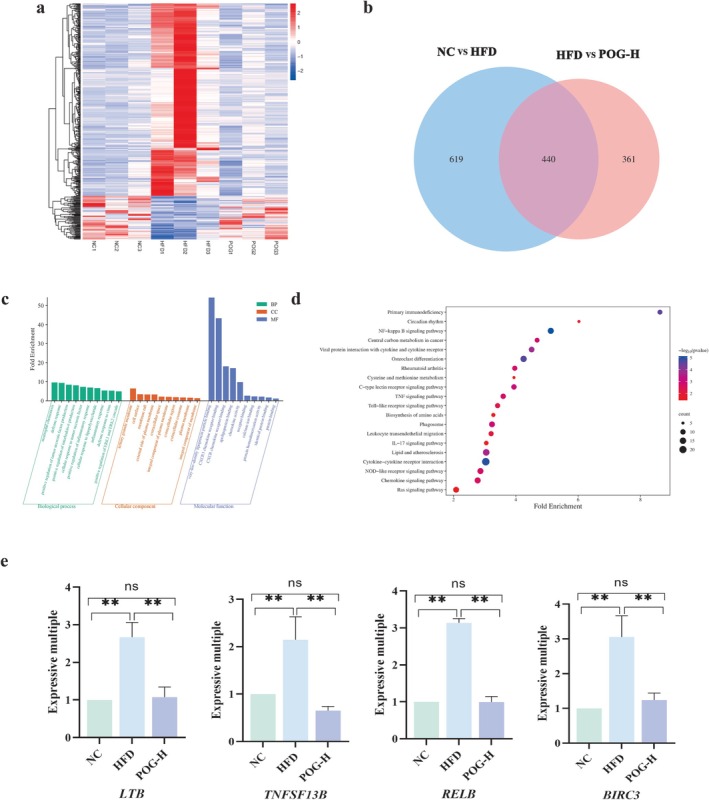
Transcriptomics analysis results of POG against HLP. (a) Heatmap of hierarchical clustering analysis of differential genes among the three groups. (b) Venn diagram of differential genes. (c) GO functional enrichment map. (d) KEGG pathway enrichment map. (e) Effect of POG on the expression of key genes.

To track the upstream variants of lipids, transcriptional profiling was performed on liver tissues from the NC group, HFD group, and POG‐H group. The results showed that compared to the NC group, the HFD group had a total of 1060 differentially expressed genes, among which 302 genes were upregulated and 758 genes were downregulated. Compared to the HFD group, the H‐POG group had a total of 803 differentially expressed genes, with 186 genes being upregulated and 617 genes being downregulated. The heatmap of differential gene expression clustering clearly displayed the expression levels of the three groups (Figure 3a). From the Venn diagram, it could be seen that there was a total of 440 overlapping genes between the two comparisons (Figure 3b). Using the David online tool, GO and KEGG analyses were conducted on the differentially expressed genes after drug administration to identify the pathways and potential mechanisms through which POG lowers lipids. The GO enrichment analysis revealed that these differentially expressed genes participate in various biological processes such as positive regulation of interleukin‐6 production, positive regulation of inflammatory response, cellular response to lipopolysaccharide, inflammatory response, very‐low‐density lipoprotein particle binding, CXCR chemokine receptor binding, apolipoprotein binding, chemokine activity, and calcium ion binding, among others (Figure 3c). In addition, KEGG analysis showed that these differentially expressed genes are significantly enriched in pathways such as NF‐kappa B signaling pathway, lipid and atherosclerosis, TNF signaling pathway, chemokine signaling pathway, Toll‐like receptor signaling pathway, IL‐17 signaling pathway, biosynthesis of amino acids, and cysteine and methionine metabolism, among others (Figure 3d).

### 3.6 Effect of POG on the Expression of Genes Related to Hepatic Lipid Metabolism

We used real‐time fluorescence quantitative PCR to identify the expression levels of the LTB, TNFSF13B, RELB, and BIRC3 genes in order to further corroborate the mechanism of action of POG on HLP. One‐way ANOVA revealed significant main effects of treatment on the expression of all four genes (LTB: *F*(2, 6) = 12.91, *p* = 0.0067; TNFSF13B: *F*(2, 6) = 7.438, *p* = 0.0237; RELB: *F*(2, 6) = 128.8, *p* < 0.0001; BIRC3: *F*(2, 6) = 39.82, *p* = 0.0003). Post hoc Tukey's test confirmed that the expression levels of all four genes were significantly higher in the HFD group compared to the NC group (all *p* < 0.05), suggesting that HFD feeding stimulates the inflammatory response, activates the NF‐κB signaling pathway, and upregulates the expression of key genes in this pathway. When comparing the POG‐treated groups to the HFD group, the expression of LTB, TNFSF13B, RELB, and BIRC3 was significantly downregulated (all *p* < 0.05). This indicates that POG can significantly inhibit the NF‐κB signaling pathway, thereby alleviating lipid metabolism abnormalities by reducing the inflammatory response (Figure 3e).

## 4 Discussion

HLP is a prevalent metabolic disorder characterized by elevated levels of LDL‐C and/or decreased levels of HDL‐C in the blood, serving as a primary risk factor for cardiovascular diseases (Grundy et al. 2019). The etiology of HLP involves both genetic and environmental factors. Genetic predispositions, such as mutations in lipid metabolism‐related genes (e.g., APOB, LDLR), contribute to hereditary lipid abnormalities (Wiegman et al. 2015), while environmental factors, including high‐fat diets, sedentary lifestyles, and excessive alcohol consumption, exacerbate metabolic imbalances (Imdad et al. 2024).

POG is a rich source of bioactive compounds, including phenolic acids, tannins, and flavonoids, which exhibit various biological activities such as antimicrobial, anticancer, antioxidant, and anti‐inflammatory effects (Eghbali et al. 2021; Hussein et al. 2021; Konstantinidi and Koutelidakis 2019; Lee et al. 2010; Morittu et al. 2020; Murthy and Bapat 2020). Phenolic acids (e.g., gallic acid) exhibit strong antioxidant activity due to their pyrogallol structure, while specifically inhibiting the NF‐κB signaling pathway to reduce TNF‐α levels. It also modulates glucose and lipid metabolism (Al‐Muammar and Khan 2012; El‐Hadary and Ramadan 2019; Jafri et al. 2000) with potential benefits for obesity (Konstantinidi and Koutelidakis 2019; Pathil et al. 2012) and insulin resistance (Gharib and Kouhsari 2019). Punicalagin, a characteristic ellagitannin, improves insulin resistance by upregulating PPARγ expression, thereby enhancing glucose metabolism. Ellagic acid demonstrates dual regulation of both inflammatory and metabolic pathways to ameliorate insulin resistance. The fruit, seeds, peel, and flowers of POG have been traditionally used to treat conditions like ulcers, inflammation, Alzheimer's disease, erectile dysfunction, cancer, cerebral ischemia, obesity, and microbial infections (Ahmed et al. 2014; Al‐Muammar and Khan 2012; Forest et al. 2007; Hussein et al. 2014; Kasimsetty et al. 2010; Subash et al. 2014; Sun, Ma, et al. 2019). Recent studies have highlighted the role of POG extracts in regulating lipid metabolism disorders, offering therapeutic benefits for related conditions (Al‐Moraie et al. 2013; Esmaillzadeh et al. 2004; Yang et al. 2018).

In this study, we employed metabolomics and transcriptomics approaches to systematically elucidate the molecular mechanisms. Our findings demonstrate that POG significantly improved the lipid profile in HLP rats, reducing TC, TG, LDL‐C, and liver injury markers while increasing HDL‐C. These results suggest that POG effectively alleviates lipid accumulation and enhances lipid homeostasis, highlighting its potential as a promising therapeutic agent for HLP.

The observed differences in body weight between the HFD and POG‐L groups, despite similar initial weights, were accompanied by significant changes in liver weight/body weight and white adipose tissue/body weight ratios. This indicates that POG treatment may exert independent effects on fat deposition and liver function, irrespective of overall weight reduction (Alami et al. 2023). Specifically, POG appeared to target key metabolic pathways, such as lipogenesis and fatty acid oxidation, without significantly affecting total body weight. This finding underscores the potential of POG to promote lipid balance and liver protection in the absence of weight loss, a critical aspect of its therapeutic efficacy in metabolic disorders (ALTamimi et al. 2022).

Our metabolomics analysis identified 37 HLP‐associated metabolites, implicating key pathways such as sphingolipid metabolism, tyrosine metabolism, pyrimidine metabolism, and arachidonic acid metabolism. Notably, alterations in bile acid metabolites, including cholesterol sulfate and glycochenodeoxycholic acid, were observed, which may contribute to improved lipid metabolism and enhanced bile excretion following POG treatment (Qi et al. 2015). These findings align with the known roles of POG polyphenols in modulating lipid metabolism and suggest that POG regulates a complex metabolic network, explaining its beneficial effects on the lipid profile.

Transcriptomics analysis further revealed significant changes in the expression of genes involved in lipid metabolism and inflammatory pathways. Of particular interest was the downregulation of the NF‐κB signaling pathway, which plays a pivotal role in inflammation (Zhao et al. 2014). This finding is consistent with the established role of chronic inflammation in promoting lipid accumulation and metabolic dysfunction. POG appears to exert its anti‐inflammatory effects by modulating the NF‐κB pathway, reducing inflammatory markers, and potentially preventing the progression of lipid‐related disorders. The intricate relationship between lipid metabolism and inflammation is well documented, and our data suggest that POG's dual action on these pathways may underlie its efficacy in treating HLP (Hotamisligil 2017).

While our study provides valuable insights into the effects of POG on HLP, several limitations should be noted. First, we did not directly measure weekly caloric intake. Although we used a HFD with known composition and caloric content, and ensured consistent diet consumption across groups, variations in caloric intake could have some effects on the results. Future studies should include direct caloric intake measurements to more comprehensively assess the impact of diet on lipid metabolism. Second, given the phosphorylation‐dependent nature of the NF‐κB pathway, we acknowledge the importance of validating this pathway through Western blot analysis. Although this was not performed in the current study, transcriptomic data and gene expression analysis provide strong evidence for NF‐κB's involvement in mediating POG's therapeutic effects (Zhou et al. 2023). Nonetheless, incorporating Western blotting in future studies will provide more direct validation of the underlying mechanisms. Additionally, while transcriptomic and PCR analyses were performed with three biological replicates, increasing the sample size in future studies will improve statistical power and the reliability of our findings (Pang et al. 2020). Lastly, while this study focused on the lipid‐lowering and anti‐inflammatory effects of POG, further research on its pharmacokinetics, bioavailability, and potential contraindications is necessary to support its development as a therapeutic agent for hyperlipidemia in clinical and dietary settings.

## 5 Conclusion

In conclusion, our study demonstrates that POG exhibits significant therapeutic potential for HLP by improving the lipid profile, reducing liver injury, and modulating inflammatory pathways. Although the results are promising, future research should include larger sample sizes, comprehensive molecular analyses, and additional techniques such as Western blotting to further elucidate the mechanisms underlying POG's beneficial effects. This work lays the foundation for developing POG‐based therapeutic strategies to manage HLP and related metabolic disorders.

## Author Contributions

**Hao Wang:** writing – original draft, methodology. **Jingtao Liu:** visualization. **Xin Dong:** supervision, data curation, methodology. **Tiantian Shi:** investigation. **Xuena Zhang:** investigation. **Yide He:** investigation. **Shaonan Hu:** investigation, formal analysis, supervision. **Yuewu Wang:** supervision, formal analysis. **Jian Zheng:** writing – review and editing, supervision. **Peifeng Xue:** writing – review and editing, resources, project administration, funding acquisition.

## Funding

This study was financially supported by the National Natural Science Foundation of China (grant number 82160745).

## Ethics Statement

All rats care and research were carried out according to the principles of the guidelines for the care and use of experimental animals and were approved by the Experimental Animal Ethics Committee of Inner Mongolia Medical University.

## Conflicts of Interest

The authors declare no conflicts of interest.

## Supporting information


**Figure S1:** Pomegranate chromatographic profile.
**Figure S2:** Punicalagin chromatographic profile.
**Figure S3:** Gallic acid chromatographic profile.
**Figure S4:** Ellagic acid chromatographic profile.

## Data Availability

The data that support the findings of this study are available from the corresponding author upon reasonable request.
