# Supplementary material for: The Effects and Mechanisms of Pomegranate on Regulating Lipid Metabolism
Source: Food Sci Nutr. 2026 Apr 15;14(4):e71789. doi: 10.1002/fsn3.71789 (PMC13082919; doi:10.1002/fsn3.71789)
Supplement: Supplementary file 1 — Figure S1: Pomegranate chromatographic profile. Figure S2: Punicalagin chromatographic profile. Figure S3: Gallic acid chromatographic profile. Figure S4: Ellagic acid chromatographic profile. [file FSN3-14-e71789-s001.docx]

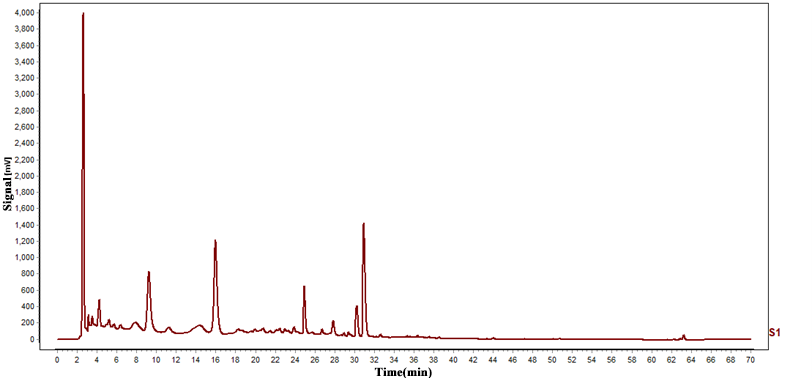


Supplementary Figure 1:Pomegranate Chromatographic Profile

(Each component content in pomegranate: Punicalagin is 311.3 μg/ml; Gallic Acid is 4.9μg/ml; Ellagic Acid is 126.6 μg/ml)


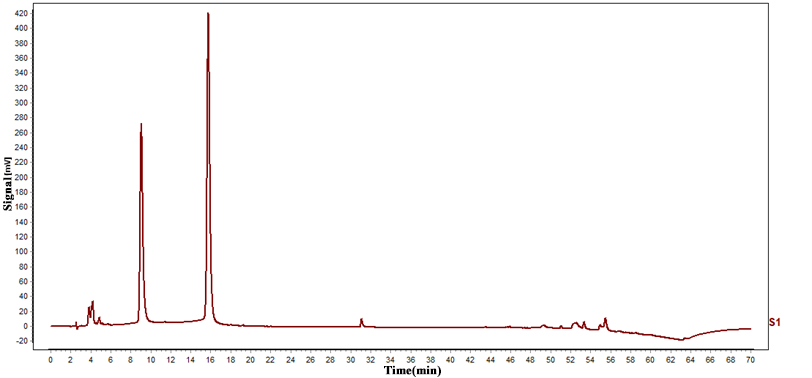


Supplementary Figure 2:Punicalagin Chromatographic Profile


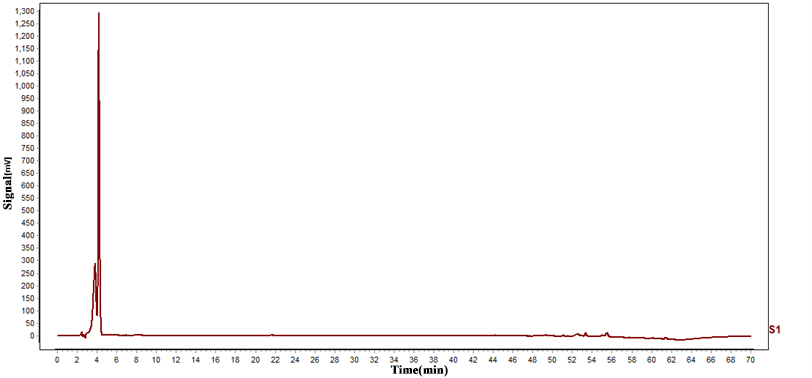


Supplementary Figure3:Gallic Acid Chromatographic Profile


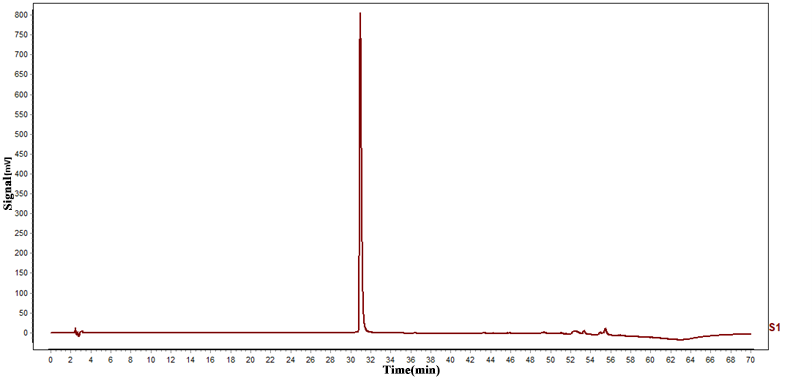


Supplementary Figure 4:Ellagic Acid Chromatographic Profile
